# Nano-Polyplexes Mediated Transfection of Runx2-shRNA Mitigates the Osteodifferentiation of Human Valvular Interstitial Cells

**DOI:** 10.3390/pharmaceutics12060507

**Published:** 2020-06-02

**Authors:** Geanina Voicu, Daniela Rebleanu, Cristina Ana Constantinescu, Elena Valeria Fuior, Letitia Ciortan, Ionel Droc, Cristina Mariana Uritu, Mariana Pinteala, Ileana Manduteanu, Maya Simionescu, Manuela Calin

**Affiliations:** 1 Institute of Cellular Biology and Pathology “Nicolae Simionescu” of the Romanian Academy, 050568 Bucharest, Romania; geanina.voicu@icbp.ro (G.V.); daniela.rebleanu@icbp.ro (D.R.); cristina.constantinescu@icbp.ro (C.A.C.); elena.fuior@icbp.ro (E.V.F.); letitia.ciortan@icbp.ro (L.C.); ileana.manduteanu@icbp.ro (I.M.); maya.simionescu@icbp.ro (M.S.); 2 Central Military Hospital “Dr. Carol Davila”, Cardiovascular Surgery Clinic, 010825 Bucharest, Romania; ionel.droc@gmail.com; 3 Centre of Advanced Research in Bionanoconjugates and Biopolymers, “Petru Poni” Institute of Macromolecular Chemistry, 700487 Iasi, Romania; uritu.cristina@icmpp.ro (C.M.U.); pinteala@icmpp.ro (M.P.); 4 Advanced Centre for Research-Development in Experimental Medicine, Grigore T. Popa University of Medicine and Pharmacy of Iasi, 700115 Iasi, Romania

**Keywords:** calcific aortic valve disease, valvular interstitial cells, Runx2, nanocarriers, shRNA, osteodifferentiation

## Abstract

Calcific aortic valve disease (CAVD) is a progressive disorder that increases in prevalence with age. An important role in aortic valve calcification is played by valvular interstitial cells (VIC), that with age or in pathological conditions acquire an osteoblast-like phenotype that advances the disease. Therefore, pharmacological interventions aiming to stop or reverse the osteoblastic transition of VIC may represent a therapeutic option for CAVD. In this study, we aimed at developing a nanotherapeutic strategy able to prevent the phenotypic switch of human aortic VIC into osteoblast-like cells. We hypothesize that nanocarriers designed for silencing the Runt-related transcription factor 2 (Runx2) will stop the progress or reverse the osteodifferentiation of human VIC, induced by high glucose concentrations and pro-osteogenic factors. We report here the potential of fullerene (C60)-polyethyleneimine (PEI)/short hairpin (sh)RNA-Runx2 nano-polyplexes to efficiently down-regulate Runx2 mRNA and protein expression leading subsequently to a significant reduction in the expression of osteogenic proteins (i.e., ALP, BSP, OSP and BMP4) in osteoblast-committed VIC. The data suggest that the silencing of Runx2 could represent a novel strategy to impede the osteoblastic phenotypic shift of VIC and the ensuing progress of CAVD.

## 1. Introduction

Calcific aortic valve disease (CAVD) is a common disorder that increases in prevalence with age or in pathological conditions. The early stage of CAVD, the aortic sclerosis affects up to 25% of people over 65 years in developed countries, whereas for the late stage, i.e., calcific aortic stenosis, the prevalence is 1.7% in the population over 65 [1]. The pathology is characterized by the thickening of the valve leaflets, due to fibrosis and calcification processes, causing impairment of valvular motion and obstruction of left ventricular blood outflow, leading to cardiac hypertrophy and ultimately, heart failure [2]. The pathogenesis of this disease includes genes and proteins implicated in the subendothelial accumulation of atherogenic lipoproteins, chronic inflammation, fibrosis, neovascularization and ectopic calcification [3,4]. The only effective treatments for aortic stenosis are surgical aortic valve replacement or the minimally invasive transcatheter aortic valve implantation (TAVI). At present, there are no other efficient pharmacological treatments to prevent or reverse CAVD [5]. Although there are numerous similarities between CAVD and atherosclerosis, the main treatment of the latter, namely the administration of statins, has no effect on CAVD progression [6]. In this context there is a major unmet clinical need to develop specific and effective therapies for CAVD.

Valvular interstitial cells (VIC) are a heterogeneous population of cells implicated in maintaining valve homeostasis [7,8]. The activation of VIC is a normal regenerative process in the valve, but in pathological conditions, i.e., hyperlipidemia, diabetes or atherosclerosis, their activation can lead to the inception of CAVD [9]. In the early phase of CAVD, the inflammation can induce a phenotypic transition of VIC, from quiescent cells to myofibroblasts, and later on, in response to pro-osteogenic stimulation (e.g., inflammation, mechanical stress or cell- extracellular matrix interaction), they could undergo an osteodifferentiation process leading to ectopic calcification of the valve leaflets [10,11,12]. A master transcription factor implicated in osteoblast differentiation is Runt-related transcription factor 2 (Runx2)/Core-binding factor 1 (Cbfa1) that regulates transcription and determines the increased expression of osteogenic genes such as collagen I, alkaline phosphatase (ALP), osteopontin (OSP), bone sialoprotein (BSP) and osteocalcin (OCN) [13,14]. Runx2 is not expressed in normal aortic valves, but its expression is induced in CAVD [15,16,17,18]. It was reported that in porcine aortic VIC treated with Klotho-deficient fetal bovine serum and cholesterol, downregulation of Runx2 with siRNA abolishes the upregulation of collagen I and osteocalcin protein expression indicating that Runx2 silencing could prevent the osteodifferentiation and matrix remodeling in VIC [19]. In another study, the treatment of C2C12 cells (mouse myoprogenitor cells), stimulated to an osteogenic phenotype, with polymer-lipid nanoparticles complexed with siRNA targeting Runx2 inhibits alkaline phosphatase activity and calcium phosphate deposition [20]. 

CAVD is accelerated in diabetes [21,22,23]. Recently, it was shown an increased expression of inflammatory and pro-osteogenic markers in the aortic valves of diabetic hyperlipemic ApoE-deficient mice as compared with non-diabetic hyperlipemic controls [24]. Moreover, elevated mRNA Runx2 level was determined in the aortic tissues of diabetic LDLr−/−/ApoB100/100/IGF-II mice as compared to non-diabetic or wild type C57BL/6 control mice [21]. Since the osteoblast differentiation of VIC leads to aortic valve calcification, developing therapeutic approaches to impede VIC phenotypic shift could lead to novel interventions to halt or reverse CAVD. Lately, RNA interference (RNAi), accomplished with small interfering RNAs (siRNAs) or short hairpin RNAs (shRNAs), has developed as a powerful approach for specific degradation of a target mRNA [25]. 

In this study, we aimed at developing a nanotherapeutic strategy able to prevent the differentiation of human aortic VIC in osteoblast-like cells in diabetic and pro-osteogenic conditions. We hypothesize that nanocarriers designed for Runx2 RNA interference could halt or reverse the osteodifferentiation of human VIC exposed to high glucose concentrations or/and osteogenic factors. Previously, we have developed dendrimer-like structures of branched low molecular weight polyethyleneimine (PEI) (2 kDa) organized around a fullerene (C60) core, able to act as gene vectors, by forming stable polyplexes with plasmid DNA [24]. For our experiments, we designed nanocarriers consisting of nano-polyplexes formed between the nano-conjugates of C60-PEI and the plasmids containing shRNA sequences specific for Runx2 (shRNA-Runx2). We report here the potential of C60-PEI/shRNA-Runx2 nano-polyplexes to efficiently down-regulate Runx2 mRNA and protein expression leading subsequently to a significant reduction in the expression of several osteogenic proteins (i.e., ALP, BSP, OSP and BMP4). 

## 2. Materials and Methods

### 2.1. Reagents

The commercial sources of the main reagents and consumables were as follows: Dulbecco’s modified Eagle’s medium (DMEM) and Runx2 MISSION shRNA Plasmid DNA (MISSION Runx2 shRNA plasmid DNA, catalog no. SHCLND-NM_004348) were from SIGMA-Aldrich (Merck KGaA, Darmstadt, Germany), fetal bovine serum (FBS), penicillin and streptomycin from Gibco (ThermoFisher Scientific, Waltham, MA, USA); cell culture dishes were from TPP^®^ (Trasadingen, Switzerland); 2,3-Bis-(2-Methoxy-4-Nitro-5-Sulfophenyl)-2H-Tetrazolium-5-Carboxanilide (XTT), rabbit polyclonal antibodies against Runx2, bone morphogenic protein 4 (BMP4), goat polyclonal antibody against α-smooth muscle actin (SMA), SuperSignal West Dura were from Thermo Fisher Scientific (Waltham, MA, USA); goat polyclonal antibodies against bone sialoprotein (BSP) and alkaline phosphatase (ALP)were from R&D Systems (Minneapolis, MN, USA); rabbit polyclonal antibody against osteopontin was from Novus Biologicals (Centennial, CO, USA); DAPI was from Santa Cruz Biotechnology (Santa Cruz, CA, USA); X-tremeGene 9 was from Roche (Basel, Switzerland); Cy3-labeled plasmid was from Mirus Bio (Madison, WI, USA). Deionized water (18.2 MΏ/cm) was obtained in house using Milli-Q system from Millipore (Watford, UK).

### 2.2. Valvular Interstitial Cells (VIC): Isolation and Culture

Human VIC were isolated from noncalcified cusps (or portions of the cusp) of aortic valves obtained from a patient who underwent surgical valve replacement, as recently described [26]. The surgery was carried out at Central Military Hospital “Dr. Carol Davila”, Cardiovascular surgery clinic, Bucharest, according to the principles outlined in the Declaration of Helsinki for experiments involving human samples [27]. Informed-consent forms was signed by the patient and his anonymity and privacy rights were respected. The study was approved by the Ethics Committee of the Institute of Cellular Biology and Pathology “Nicolae Simionescu”.

Cultured VIC, at passages three to eight, were grown to confluence in DMEM, supplemented with 10% fetal bovine serum, 50 µg/mL neomycin, 100 UI/mL penicillin and 100 µg/mL streptomycin, in 1% gelatin-coated plates, at 37 °C, in a humidified 5% CO_2_ incubator. The cells were checked for Mycoplasma contamination, employing both a PCR assay using primers for different Mycoplasma species and a bioluminescent assay (MycoAlert mycoplasma detection kit from Lonza, Basel, Switzerland) and were found negative.

### 2.3. Preparation of Fullerene (C60)-PEI/Short Hairpin (sh)RNA Plasmid Nano-Polyplexes

To downregulate the mRNA expression of Runx2 we used, as nanocarriers for intracellular delivery of plasmids containing shRNA sequences specific for Runx2, the synthesized fullerene (C60)-polyethyleneimine (PEI) nano-conjugates as described in [28]. The C60-PEI nano-conjugates consisting of dendrimer structures with C60 as the core and bearing branched PEI arms (~2 kDa) are efficient vectors for plasmid DNA transfection [28]. To obtain C60-PEI based nano-polyplexes, C60-PEI were complexed with MISSION^®^shRNA Plasmid DNA either targeting human Runx2 gene or a control plasmid, MISSION^®^ pLKO.1-puro Non-Mammalian shRNA Control Plasmid DNA at different N/P ratio (15, 20, 25, 30 and 40), 1:1 volumes, for 30 min at room temperature. N/P ratio represents the ratio of nitrogen atoms in C60-PEI to phosphorus atoms in DNA and were calculated using the nitrogen percentage resulted from elemental analysis XPS of C60-PEI (16.6% N) [28].

### 2.4. Characterization of Nano-Polyplexes

#### 2.4.1. Size and Zeta Potential

The size of polyplexes was determined by dynamic light scattering (DLS) method using a Zetasizer Nano ZS (ZEN 3600, Malvern Instruments, Malvern, UK). The size Standard Operating Procedure (SOP) measured the scattered light intensities at an angle of 173°, using water as dispersant, at 25 °C. The Zeta potential was determined by electrophoretic light scattering (ELS), by running three consecutive measurements at 5 Volts with 300 s delay between measurements, using a Zeta dip cell (ZEN 1002) immersed into the sample. The results were analyzed using the build-in Zetasizer Software 7.12 (Malvern Instruments, Malvern, UK).

#### 2.4.2. Agarose Gel Retardation Assay

The complexation efficacy of shRNA plasmid by C60-PEI nanoconjugates at different charge ratios was investigated by electrophoresis in 1% agarose gel, stained with Midori Green Advanced, using Tris-Acetate-EDTA buffer (40 mM Tris–HCl, 1% acetic acid, 1 mM EDTA), as described elsewhere [29]. The samples were mixed with 6x Loading Buffer (0.05% Orange G, 30% glycerol) and loaded into gel. Electrophoresis was performed at 70 V for 20 min and DNA bands were visualized using an UV transilluminator.

#### 2.4.3. Evaluation of Nano-Polyplexes Cytotoxicity

Cytotoxicity was assessed using XTT (2,3-bis-(2-methoxy-4-nitro-5-sulfophenyl)-2H-tetrazolium-5-carboxanilide) Cell Proliferation Kit (Thermo Fisher Scientific). The method quantifies the reduction of XTT by NADH produced by mitochondria in metabolically active cells. VIC were plated at 5.000 cells/well in a 96-well plate and incubated for 24 h. Polyplexes formed of C60-PEI complexed with control shRNA plasmid at a fixed concentration of 0.1 µg/well and made at different charge ratios N/P = 10, 15, 20, 25, 30 were used. After 48 h of incubation with polyplexes, the cells were incubated with a mixture of XTT and PMS (Phenazine methosulfate) dissolved in colorless culture medium for 2 h, at 37 °C and 5% CO_2_, until the color turned orange. The absorbance was measured at 450 nm using TECAN Infinite M200Pro (Tecan Group Ltd., Männedorf, Switzerland). The results were normalized to control (untreated cells) and were expressed as mean ± S.D. (standard deviation) of three experiments made in triplicates. 

#### 2.4.4. Uptake of Nano-Polyplexes by VIC

The cells were plated in 48-well plates at a density of 25.000 cells/well. After 24 h the cells were incubated for 4 h at 37 °C with polyplexes made of C60-PEI and a Cy3-labelled plasmid at N/P = 25 or with free Cy3-labelled plasmid at a concentration of 0.3 µg/well. After microscopic examination, the cells were collected with 2.5‰ trypsin and resuspended in FACS buffer (0.5% PFA in PBS) for flow cytometry determinations (Gallios, Beckman Coulter). Data were analyzed using Flowing Software version 2.5.1.

#### 2.4.5. Transfection Assay

VIC were seeded at a density of 25.000 or 50.000 cells/well in 24-well plates. After 24 h, VIC were incubated with polyplexes formed at N/P = 15, 20, 25 between C60-PEI and the pEYFP plasmid, coding for the yellow fluorescent protein. A quantity of 1 µg pEYFP plasmid was used per well. Fluorescent protein expression was evaluated at 48 h post-transfection using a fluorescence microscope (Olympus IX81 microscope equipped with FITC filter). Also, a commercial vector, X-tremeGene 9 (Roche catalog no. 06365809001) was used for transfection according to the manufacturer instructions. 

### 2.5. Assessment of the Expression of Osteogenic Proteins in VIC Exposed to Medium Containing High Glucose Concentrations in the Absence or Presence of Osteogenic Factors

#### 2.5.1. Western Blot Assays

VIC were seeded in 6-well plates at a density of 200.000 cells/well and after 24 h were further incubated in the culture medium containing: (1) normal glucose concentration (5.5 mM); (2) high glucose (HG), 25 mM glucose concentration: (3) osteogenic medium (OM), consisting in normal, 5.5 mM glucose and osteogenic factors (50 µg/mL ascorbic acid, 10 mM β-glycerophosphate, 10 nM dexamethasone) and (4) medium containing high, 25 mM glucose and osteogenic factors (HGOM). The cells were exposed to the above conditions for different periods (2, 7, 14 and 21 days) and the medium was refreshed every two days. At the end of the incubation period, the cells were washed with cold PBS, lysed into Sx2 Laemmli buffer and protein concentration was measured by Amido Black assay. Cell protein extracts (30 µg/lane) were subjected to 12% SDS-PAGE and transferred onto nitrocellulose membranes using a Trans Blot Semi-Dry system. The blots were probed overnight with the following appropriate primary antibodies: goat anti-αSMA (1:1000, Thermo Fisher Scientific cat. no. PA5-18292), rabbit anti-Runx2 (1:200, Thermo Fisher Scientific cat. no.PA141519), goat anti-ALP (1:200, R&D Systems cat. no. AF2910), goat anti-BSP (1:1000, R&D Systems, cat. no. AF4014), rabbit anti-osteopontin (1:1000, R&D Systems cat. no. NB600-1043) and rabbit anti-BMP4 (1:1000, Thermo Fisher Scientific cat. no. PA5-27288). After thorough washing, the membranes were incubated (one hour, room temperature) with secondary antibodies, goat anti-rabbit IgG or rabbit anti-goat IgG conjugated with horseradish peroxidase (HRP, 1:1000, Thermo Fisher Scientific, cat. no. 32460 and 81-1620, respectively). After washing, the membranes were incubated with SuperSignal West Dura chemiluminescent substrate (Thermo Fisher Scientific cat. no. 34076) and visualized with ImageQuant Las 4000. The bands were quantified by densitometry using Image J program developed at the National Institutes of Health (NIH), USA. The results were normalized to β-actin, then calculated as fold change versus Control for day 2 or as fold change versus expression at day 2 for the other time intervals (7, 14 and 21 days). The data were expressed as mean ± S.D. (standard deviation) of two experiments performed in duplicates. 

### 2.6. Treatment of VIC with Nano-Polyplexes Carrying shRNA Sequences Specific for Runx2

#### 2.6.1. Transfection of VIC with C60-PEI/shRNA-Runx2 Nano-Polyplexes

MISSION^®^shRNA Plasmid DNA, purchased from Sigma-Aldrich (Merck KGaA, Darmstadt, Germany), consisted of shRNA sequences, targeting human Runx2 gene, cloned into the pLKO.1-puro vector. Three shRNA sequences specific for human Runx2, namely the clones from The RNAi Consortium (TRC) Version 1 library: TRCN0000013653 (sh_1), TRCN0000013655 (sh_2), TRCN0000013656 (sh_3) were tested for their silencing efficiency in VIC exposed to HGOM using the nano-polyplexes. MISSION^®^ pLKO.1-puro non-mammalian shRNA control plasmid DNA was validated by the manufacturer to have no homology to known mammalian genes and used as a negative control for RNA knockdown. The shRNA plasmids were amplified in Escherichia coli host strain DH5α, and subsequently isolated and purified using GenElute-Plasmid Midiprep kit (Sigma-Aldrich, Germany), according to manufacturer’s protocol. 

To obtain nano-polyplexes, C60-PEI were combined at a charge ratio N/P of 25 (1:1 volumes) with each plasmid, separately or with a mix of the three plasmids shRNA-Runx2 in equal amounts by incubation for 30 min at room temperature, under mixing. 

VIC were seeded at a concentration of 50.000 cells/well in 24-well plates and after 24 h were exposed for five days to normal medium (C) or HGOM. The medium was refreshed every 2 days. In the fifth day, VIC were transfected with nano-polyplexes formed between C60-PEI and each of the three shRNA-Runx2 plasmid (sh_1, sh_2 and sh_3) or with a mix of them. As control, MISSION^®^ pLKO.1-puro Non-Mammalian shRNA Control Plasmid DNA was used. A N/P = 25 ratio, at a concentration of 1 µg plasmid DNA/well was employed.

#### 2.6.2. Quantitative Real-Time Polymerase Chain Reaction

At 48 h after incubation of VIC with nano-polyplexes, total cellular RNA was isolated using TRIzolTM reagent according to the manufacturer’s instructions. The RNA concentration was determined with the Spectrophotometer NanoDrop ™ 1000 (Thermo Fisher Scientific, Waltham, MA, USA). The synthesis of cDNA was performed employing 1 µg total RNA and MMLV reverse transcriptase according to the manufacturer’s protocol (Invitrogen, Thermo Fisher Scientific, Waltham, MA, USA). Quantification of mRNA was done after amplification of cDNA using a LightCycler 480 Real-Time PCR System from Roche (Basel, Switzerland), SYBR Green I chemistry and primers for human Runx2 gene. The optimized amplification conditions were 2.5 mM MgCl_2_, annealing at 60 °C, and extension at 72 °C for 42 cycles. The Runx2 expression was normalized to β-actin expression and fold changes were calculated, relative to HGOM condition, using the 2−ΔΔCT method [30]. The primers used were: for human Runx2 (Ref_SeqNM_001024630.4), forward: CCGCCTCAGTGATTTAGGGC, and reverse: GGGTCTGTAATCTGACTCTGTCC, resulting in a 132 bp amplicon, and for human ACTB (Ref_Seq NM_001101.4) forward: GACGAGGCCCAGAGCAAGAGAGG, and reverse: CATGGCTGGGGTGTTGAAGGTCTC with a 231 bp corresponding amplicon.

#### 2.6.3. Western Blot Assay

Human VIC were cultured in a 6-well plate at a density of 200.000 cells/well. After 24 h, VIC were exposed either to normal medium (controls, C) or HGOM for 5 days, with a medium refresh every 2 days. Then, the cells grown in HGOM were transfected with polyplexes of C60-PEI complexed with each of the three plasmids containing shRNA sequences specific for Runx2 or with a mix of the plasmids, at a final concentration of 5 µg DNA per well (N/P = 25). Free mix of plasmids and C60-PEI/shRNA control plasmid DNA polyplexes were used as negative controls. After 48 h, VIC were processed for Western blot assay as described above (Section 2.5.1). The results were normalized to β-actin, then calculated as fold change versus HGOM (considered as 1) and expressed as mean ± S.D. (standard deviation) of three experiments performed in duplicates.

#### 2.6.4. Determination of Alkaline Phosphatase (ALP) Activity in VIC after Transfection with C60-PEI/shRNA-Runx2 Nano-Polyplexes

##### Quantification of Alkaline Phosphatase Activity

To investigate whether the reduction of Runx2 expression has an impact on osteogenic differentiation of VIC, we measured the ALP activity in VIC exposed to HGOM. The cells were seeded on 1% gelatin-coated 24-well plates (35.000 cells/well). After 24 h, the cells were exposed to normal (C) and HGOM media, for 7, 14 and 21 days, with medium changing every 2 days. In the 5th and 12th day, the cells activated with HGOM were incubated with polyplexes formed between C60-PEI and plasmids containing shRNA-Runx2 sequences codes sh_1, sh_2 and sh_3 or with a mix of the three plasmids (N/P = 25, 1 µg shRNA plasmid DNA/well). As control, a free mix and polyplexes C60-PEI/control shRNA plasmid were used. At day 7, 14 and 21, the cells were enzymatically processed for ALP assay using SensoLyte pNPP Alkaline Phosphatase Assay Kit (AnaSpec, Inc. Fremont, CA, USA) as directed by the manufacturer. The ALP activity was measured using para-nitrophenyl phosphate (pNPP) as substrate, after 30 min of incubation at room temperature, reading the absorbance at 405 nm with a spectrophotometer (TECAN Infinite M200Pro, Tecan Group Ltd., Männedorf, Switzerland). The values of ALP activity were normalized to total protein concentration determined in cell lysates.

##### Alkaline Phosphatase Staining

VIC were cultured in 24-well plates (35.000 cells/well) on 1% gelatin-coated glass coverslips for 24 h, and then were incubated in normal medium (C) and HGOM, with medium changing every 2 days. On the 5th and 12th day of VIC exposed to HGOM, two transfections using C60-PEI/shRNA-Runx 2 plasmids (N/P = 25, 1 µg shRNA plasmid DNA/well) were performed. As controls, a free mix of plasmids and polyplexes C60-PEI/control shRNA plasmid were used. In the 14th day, the cells were processed for ALP staining, as previously described [31]. Briefly, VIC were washed twice with PBS and fixed with cold acetone for 5 min at −20 °C. Then, the cells were incubated (60 min at 37 °C) with a solution made by mixing 10 mL sodium 5,5-diethylbarbiturate (2%), 10 mL β-sodium glycerophosphate (3%), 20 mL CaCl2 (2.7%), 1 mL MgSO_4_ × 7H_2_O (5%) and 5 mL distilled H2O, pH = 9.4. Next, the cells were washed twice with alkaline water (5 min) and incubated with 2% cobalt nitrate for 5 min. After washing with distilled water and incubation with 0.5% ammonium polysulfide until a black precipitate has formed, VIC washed with distilled water and mounted on a microscope slide were examined with Olympus IX81 microscope.

### 2.7. Statistical Analysis

The results were expressed as mean ± standard deviation (S.D.) and experiments were performed in duplicate or triplicate. Statistical evaluation was carried out by one-way ANOVA with multiple comparisons post-hoc Tukey test using GraphPad Prism 7 software. Differences were considered to be statistically significant when *p* < 0.05.

## 3. Results

### 3.1. Characterization of Nano-Polyplexes

#### 3.1.1. Size and Zeta Potential

**Figure 1:**
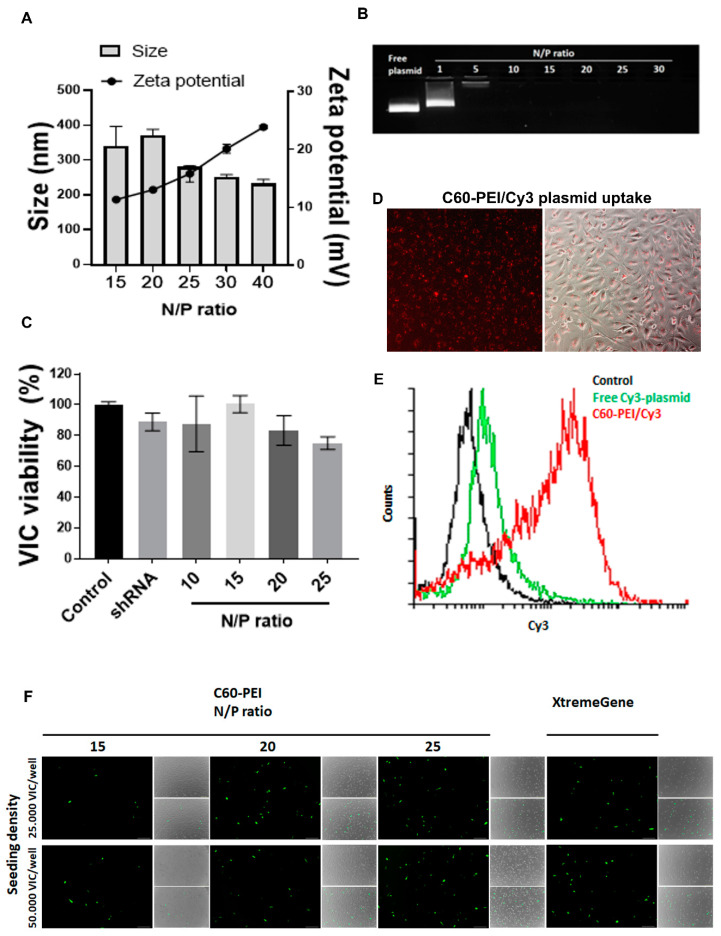
Characterization of fullerene (C60)-polyethyleneimine (PEI)/short hairpin (sh) RNA plasmid nano-polyplexes. (**A**) Average hydrodynamic diameter and ζ-potential of C60-PEI/shRNA plasmid polyplexes at different N/P ratios. Results are reported as mean ± S.D. for three individual measurements. (**B**) Agarose gel retardation assay performed for free shRNA plasmid and C60-PEI/shRNA plasmid polyplexes at different N/P ratios (200 ng shRNA plasmid/lane). (**C**) Viability of VIC exposed for 48 h to different N/P ratios of C60-PEI/shRNA plasmid nano-polyplexes. Data are presented as mean ± S.D. of three experiments made in three replicates (n = 9). * *p* < 0.05 and *** *p* < 0.001 versus control cells. (**D**,**E**) Uptake of C60-PEI/Cy3-labeled plasmid nano-polyplexes (N/P = 25) by VIC, observed by fluorescence microscopy (**D**) and by flow cytometry analysis (**E**). (**F**) Expression of fluorescent protein in VIC transfected with C60-PEI/pEYFP plasmid at N/P ratio of 15, 20 and 25 or with a commercial transfection reagent, as revealed at 48 h after transfection by fluorescence microscopy (scale bar 200 µm).

The average hydrodynamic diameter and ζ-potential of nano-polyplexes C60-PEI/shRNA plasmid formed at different N/P ratio were measured after 1:1000 dilution in distilled water. A decrease in the size of nano-polyplexes was determined as the N/P ratio increased: thus at N/P = 15 and N/P = 20, the size was around 350 nm, whereas at N/P = 25, 30 and 40, the size was about 250 nm (Figure 1A). 

The ζ-potential of nano-polyplexes was positive and increased as N/P ratio augmented. Thus, its value is ~ +10 mV at N/P = 15, ~ +15 mV at N/P = 25, reaching ~ +20 mV at N/P = 30 and ~ +25 mV at N/P = 40 (Figure 1A).

#### 3.1.2. Nano-Polyplexes Effectively Packs shRNA Plasmid

Agarose gel mobility shift assay was employed to assess the reduction of shRNA plasmid DNA electrophoretic mobility as a consequence of condensation with cationic carriers. The migration of nano-polyplexes in gel was impeded when the DNA was completely packed by nano-carrier. Free shRNA plasmid DNA or nano-polyplexes formed at 1, 5, 10, 15, 20, 25 and 30 N/P ratio (with the amount of 200 ng DNA/lane) were loaded in a 1% agarose gel containing Midori-Green and the plasmid lanes were visualized under UV light (Figure 1B). Compared to free plasmid, the migration of shRNA plasmid DNA was completely blocked at N/P higher or equal with 5. Moreover, at N/P ≥ 10, no staining was observed in the loading channel, suggesting that starting with N/P = 10, the shRNA plasmid is tightly packed by C60-PEI and DNA staining agent cannot infiltrate and bind to DNA.

#### 3.1.3. Cytotoxicity of C60-PEI/shRNA Plasmid Nano-Polyplexes

The viability of VIC exposed for 48 h to different N/P ratios of C60-PEI/shRNA plasmid polyplexes was determined by XTT assay. The data were normalized and presented as percent from control cells, cultured in the absence of nano-polyplexes, considered 100% viable (Figure 1C). The results revealed that the viability of VIC was not affected by the incubation with C60-PEI/shRNA plasmid at various N/P ratio. Also, the exposure of VIC to free shRNA plasmid, at concentration used to form nano-polyplexes (0.1 µg /well) did not affect cellular viability. The data show that C60-PEI/shRNA plasmid polyplexes are cyto-compatible and can be further used in transfection experiments. 

#### 3.1.4. C60-PEI/Cy3-Labelled Plasmid Nano-Polyplexes Are Efficiently Taken up by VIC

Fluorescence microscopy images (Figure 1D) and flow cytometry data (Figure 1E) revealed that C60-PEI/Cy3-labelled plasmid nano-polyplexes are taken up efficiently by VIC. The mean fluorescence intensity in VIC incubated with C60-PEI/Cy3-labelled plasmid was three times higher than that measured in cells incubated with the same quantity of free Cy3-labelled plasmid (Figure 1E).

#### 3.1.5. The Transfection Efficiency of Nano-Polyplexes in VIC

The highest rate of transfection, assessed by the expression of fluorescent protein, was determined in VIC seeded at a cellular density of 50.000 cells/well, at 48 h after transfection with C60-PEI/pEYFP plasmid polyplexes at N/P ratio of 25 (Figure 1F). We found that, although the fluorescent protein is expressed, the transfection reagent X-tremeGene 9 (at 6:1 ratio) had a cytotoxic effect on VIC (reduced number of cells seen in phase contrast images) as compared with highest N/P ratio of C60-PEI/pEYFP plasmid polyplexes. The results showed that the C60-PEI nanoconjugates are efficient vectors for intracellular delivery of a plasmid in VIC.

### 3.2. Time Course Expression of Osteogenesis-Related Markers in VIC Exposed to High Glucose Concentrations in the Absence or Presence of Osteogenic Factors

**Figure 2:**
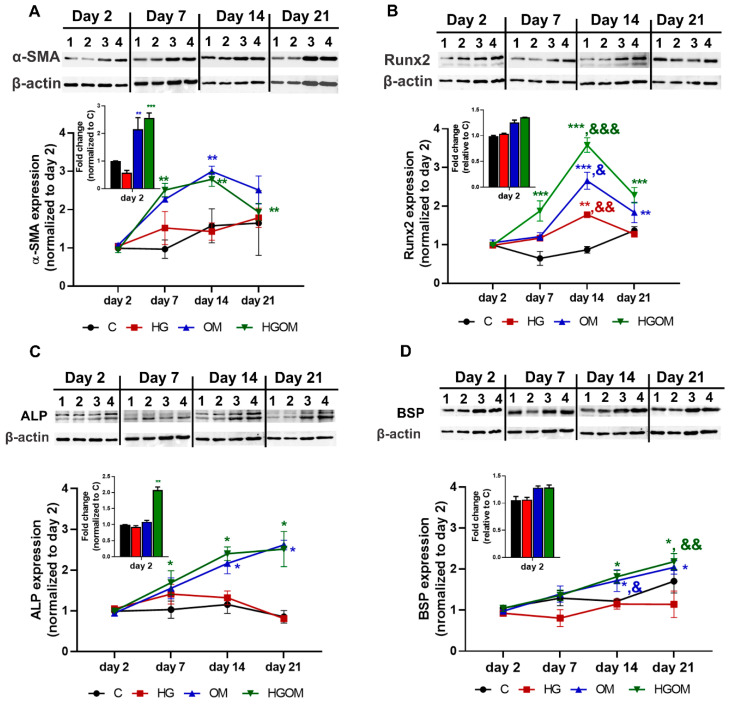
Time course expression of osteogenesis-related markers in valvular interstitial cells (VIC) exposed for 2, 7, 14 and 21 days to C (lane 1), high glucose concentration (HG) (lane 2), osteogenic medium (OM) (lane 3) or a combination of HG and OM (HGOM) (lane 4). The level of protein expression of α-smooth muscle actin (α-SMA) (**A**), runt-related transcription factor 2 (Runx2) (**B**), alkaline phosphatase (ALP) (**C**) and bone sialoprotein (BSP) (**D**) was determined by Western blot for different activation conditions, quantified by reporting to β-actin level at different periods and normalized to the day 2 values. Inserts show the level of protein expression at day 2 expressed as fold induction over control cells exposed to 5 mM glucose. Results represent the means ± S.D. of three independent experiments performed in duplicate (n = 6). Representative blots are shown above the graphs. * *p* < 0.05, ** *p* < 0.01, *** *p* < 0.001 versus values determined at day 2. & *p* < 0.05, && *p* < 0.01. &&& *p* < 0.001 versus values determined at day 7; Inserts: * *p* < 0.05, ** *p* < 0.01, *** *p* < 0.001 versus control.

To investigate the effect of high glucose (mimicking diabetes) and osteogenic factors on VIC activation and osteogenic differentiation, the cells were incubated with medium containing 5.5 mM glucose (C), or 25 mM high glucose (HG), or osteogenic factors (50 µg/mL ascorbic acid, 10 mM β-glycerophosphate, 10nM dexamethasone) in the presence of 5.5 mM glucose (OM) or 25 mM glucose (HGOM). VIC were cultured for 2, 7, 14 and 21 days in C, HG, OM and HGOM and the protein expression levels of myofibroblast differentiation marker, α-SMA and of osteogenic markers, namely Runx2, ALP and BSP were determined by Western blot assay (Figure 2). 

The level of α-SMA protein expression in VIC grown on gelatin-coated plates and exposed to medium containing normal glucose concentrations (C) did not vary significantly with the time of incubation (Figure 2A). Exposure of VIC to HG for 2 days induced a significant decrease (~30%) of α-SMA protein expression as compared to C, but at the other intervals, the α-SMA expression in HG-treated cells was found similar with that of control cells. The expression of α-SMA in OM and HGOM – exposed VIC quantified at day 2, was similar for both type of activation namely ~2 - 2.5-fold increase as compared to control (Figure 2A, insert). The time-course determination of protein expression in VIC cultured in OM and HGOM revealed that α-SMA expression increased at day 7 and day 14 (~2.5 fold versus day 2), followed by a decrease at day 21 (Figure 2A). Yet, the α-SMA level was ~2-fold higher that the level measured at day 2. No statistically significant differences were determined between the levels of α-SMA protein expression in VIC incubated in OM and HGOM, at each time point. In another series of experiments, we found that the protein expression of Runx2, the transcription factor with a key role in osteogenic differentiation, was modulated in time by the exposure of VIC to HG, OM or HGOM (Figure 2B). Two days of exposure to high glucose concentration together with osteogenic factors (HGOM) determined a significant increase in Runx2 levels (~1.5 fold) when compared to C (Figure 2B, insert). A significant increase of ~1.5-fold in Runx2 protein expression was determined in VIC exposed to HG for 14 days, when compared to Runx2 expression at 2 days, whereas at 21 days the Runx2 protein level was similar with that measured in cells grown in control medium for 21 days. Treatment of VIC with HGOM determined a significant (2-fold) increase in Runx2 levels at day 7. This increase was significantly higher when compared with Runx2 expressions in VIC treated with OM for 7 days. The time-course experiments showed a peak of Runx2 expression at 14 days in cells incubated in OM and HGOM with an increase by ~2.5-fold and ~3.5-fold, respectively determined when the Runx2 levels were normalized to the level measured at day 2. By the end of day 21, Runx2 expression decreased slightly; nevertheless, the level in OM and HGOM-treated cells remained ~2-fold above the level determined at day 2 and significantly higher as compared to HG (*p* < 0.001 and *p* < 0.01, respectively) (Figure 2B). These results suggested that the combination of high glucose concentrations with osteogenic factors has a synergistic effect on Runx2 levels determining a significant increase above the values obtained when high glucose or OM alone were employed. 

The expression of two osteogenic proteins, ALP and BSP, the earliest sign of cell calcification exhibited a time-dependent increase for both OM and HGOM treatment conditions (Figure 2C,D). The protein expression of these calcification markers was higher in HGOM-treated VIC when compared to HG, clearly showing that high glucose concentration was not sufficient to increase the level of osteogenic proteins in VIC, and the exposure to OM or a combination of high glucose concentration with osteogenic factors was necessary. Thus, the increases in ALP and BSP expression in VIC cultured in both type of activation medium, OM and HGOM, normalized at values obtained at day 2, were of about 2.5-fold (for ALP) at 14 and 21 days and ~ 2-fold on day 21 (for BSP) (Figure 2C,D).

### 3.3. In VIC, C60-PEI/shRNA-Runx2 Nano-Polyplexes Down-Regulate Runx2 Gene and Protein Expression

Thus far, our experiments showed that C60-PEI-based nano-polyplexes are efficiently taken up by VIC and the expression of the fluorescent protein encoded by the delivered plasmid, pEYFP, is induced in VIC at 48 h after transfection with C60-PEI/pEYFP polyplexes (Figure 1D–F). Our next inquiry was to determine whether the increased cellular uptake and the fluorescent protein expression are translated also in gene silencing by RNA interference. To this purpose shRNA sequences specific for Runx2, namely sh_1, sh_2 and sh_3, were delivered to human cultured VIC using C60-PEI/shRNA-Runx2 plasmids. 

Since compared to other stimulating conditions (HG and OM), the exposure of VIC to HGOM for up to 7 days determined the increased expression of myofibroblast activation marker, α-SMA and the earliest marker of osteogenesis, Runx2, we choose, for further experiments of Runx2 silencing, to expose the cells to HGOM for 5 days before transfection with shRNA-Runx2 plasmids and to assess Runx2 expression at 2 days after transfection.

**Figure 3:**
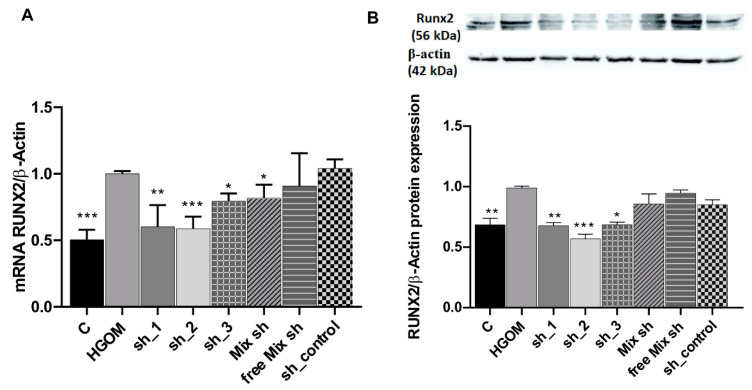
Down-regulation of Runx2 mRNA (**A**) and protein (**B**) expression in HGOM-treated VIC and transfected with C60-PEI/shRNA-Runx2 plasmids polyplexes containing different shRNA sequences specific for Runx2 (sh_1, sh_2 and sh_3) and then investigated at 48 h after transfection. Results, normalized to β-actin, were expressed as mean ± S.D. of three experiments made in duplicate (n = 6) and represent fold change relative to HGOM condition (considered as 1). * *p* < 0.05, ** *p* < 0.01, *** *p* < 0.001 compared with HGOM.

Real-time PCR experiments showed a 2-fold increase in Runx2 mRNA levels in VIC exposed for 7 days to HGOM as compared to C. The data validated the down regulation of Runx2 by shRNA sequences specific for Runx2 delivered by C60-PEI/shRNA plasmid nano-polyplexes (Figure 3A). Transfection of VIC with sh_1 and sh_2 determined a similar level of down-regulation (~40%, *p* < 0.01 and *p* < 0.001) of Runx2 mRNA. When the sh_3 plasmids or the mix of the three (sh_1, sh_2, and sh-3) plasmids were used, the down regulation of Runx2 mRNA was about 20% (*p* < 0.05) (Figure 3A). No reduction in Runx2 mRNA was detected when polyplexes C60-PEI/shRNA were used as control. 

Western blot assays revealed that the transfection of VIC exposed to HGOM with nano-polyplexes C60-PEI/shRNA-Runx2 plasmids, namely sh_1, sh_2 and sh_3 plasmids determined a significant reduction of Runx2 protein expression by ~30% (*p* < 0.01), ~40% (*p* < 0.001) and ~ 30% (*p* < 0.05), respectively, of the levels measured in HGOM-treated VIC (Figure 3B). The use of a free mix of the three shRNA plasmids and the use of a sh_control plasmid did not affect the level of Runx2 protein, which was similar to that determined in VIC exposed to HGOM.

### 3.4. In VIC Exposed to HGOM, Down-Regulation of Runx2 Expression Diminishes the Expression of Osteoblast Differentiation Markers

Having proved that the reduction of Runx2 expression at mRNA and protein level is feasible in VIC using nano-polyplexes containing shRNA-Runx2 specific sequences, we subsequently questioned whether the down regulation of Runx2 is sufficient to impede the osteoblastic differentiation of VIC exposed to HGOM. Thus, we determined the expression of osteogenic proteins, namely ALP, OSP, BSP and BMP-4 in VIC exposed for 5 days to HGOM and transfected with sh_1, sh_2 and sh_3 RNA sequences specific for Runx2 using C60-PEI nanoconjugates as transfection vectors. The expression of osteogenic proteins was ascertained at 48 h after cell transfection, by Western blot assay. 

**Figure 4:**
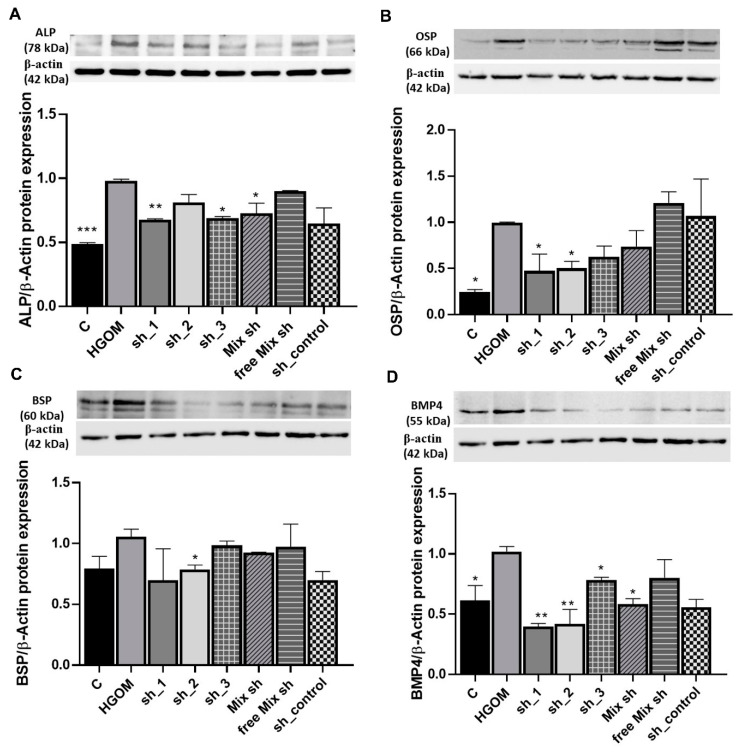
Down-regulation of Runx2 expression by C60-PEI/shRNA-Runx2 plasmids polyplexes reduces the expression of osteoblast differentiation markers ALP (**A**), OSP (**B**), BSP (**C)** and BMP-4 (**D**) in VIC exposed to HGOM. Results, normalized to β-actin, represent the means ± S.D. of three independent experiments performed in duplicate (n = 6) and represent fold change relative to HGOM condition (considered as 1). Representative blots are shown above the graphs. * *p* < 0.05, ** *p* < 0.01, *** *p* < 0.001 versus HGOM.

The results showed that the down regulation of Runx2 in HGOM-stimulated VIC reduced significantly the expression of ALP by ~30% for sh_1 and sh_3 (*p* < 0.01 and *p* < 0.05) (Figure 4A). Transfection with a mix of the three shRNA-Runx2 sequences determined also a reduction in ALP level by ~25% (*p* < 0.05). The transfection of HGOM-treated VIC with a free mix of the shRNA-Runx2 plasmids or shRNA control plasmid containing a scrambled sequence of nucleotides that do not recognize any known mammalian mRNA (sh_control) did not significantly impact the ALP protein level.

The protein level of OSP, was significantly increased (~75%) by the exposure of VIC to HGOM for 7 days as compared to C (*p* < 0.05). However, the OSP protein was significantly down regulated by ~ 50% (*p* < 0.05) when using sh_1 and sh_2 sequences specific for Runx2 (Figure 4B). When the free mix of the three shRNA-Runx2 plasmids were employed, there was no change in protein level of OSP.

For BSP, at 7 days of exposure of VIC to HGOM, no statistically significant difference in its expression compared to C was found (Figure 4C). Yet, the down regulation of Runx2 led to a decrease in BSP expression by ~ 25% in VEC transfected with C60-PEI/sh_2 plasmid polyplexes. 

The Runx2 down-regulation by RNA interference determined the reduction of the BMP-4 protein level, as well. The expression of the latter was significantly increased by 40% (*p* < 0.05) in VIC exposed to HGOM for 7 days as compared to C (Figure 4D). The transfection of VIC with C60-PEI based polyplexes formed using sh_1 and sh_2 shRNA sequences specific for Runx2 induced a significant decrease (~60%, *p* < 0.01) in BMP-4 protein level in VIC grown in HGOM. When sh_3 and a mix of the three shRNA-Runx2 plasmids were employed to form nano-polyplexes with C60-PEI, a significant reduction of ~20% and ~40% (*p* < 0.05), respectively in BMP-4 protein level was determined (Figure 4D). No statistically significant reduction in BMP-4 expression was found when a free mix of shRNA-Runx2 plasmids or sh_control plasmid was used. 

### 3.5. Reduction of Runx2 Expression Mitigates the Alkaline Phosphatase Activity in VIC Exposed to HGOM

**Figure 5:**
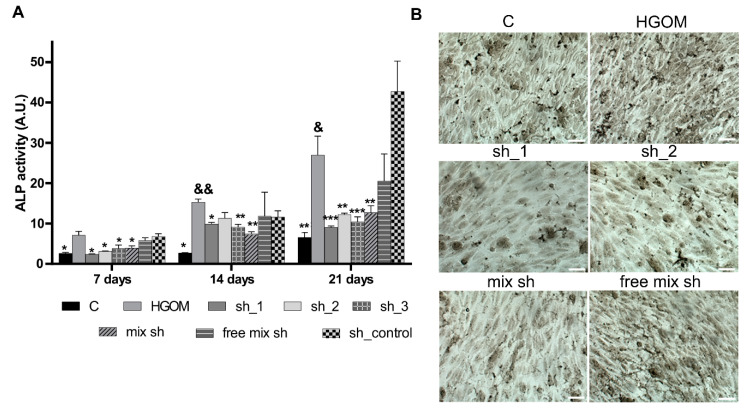
Down-regulation of Runx2 expression using C60-PEI/shRNA-Runx2 plasmids inhibits ALP activity in VIC exposed to HGOM. Quantitative assay at 7, 14 and 21 days (**A**) and histochemical detection at 14 days (**B**) of ALP activity (scale bar 100 µm). Data are presented as mean ± S.D. of two experiments made in three replicates (n = 6). * *p* < 0.05, ** *p* < 0.01, *** *p* < 0.001 compared to HGOM; & *p* < 0.05, && *p* < 0.01 versus HGOM at day 7.

As shown in Figure 5A, our experiments revealed that the enzymatic activity of ALP increased with time and was significantly higher in VIC exposed to HGOM as compared to C, at each investigated time point (7, 14 and 21 days). The increase of ALP activity determined by exposure of VIC for different intervals, to HGOM was significantly reduced by transfection with nano-polyplexes C60-PEI/shRNA-Runx2 plasmids (Figure 5A). No statistically significant inhibition was obtained when a free mix of plasmids or polyplexes C60-PEI/shRNA control were employed. At 7 days of exposure to HGOM and 48 h after transfection, a reduction of ALP activity by 66%, 56%, 46%, and 45% were obtained for transfection of VIC with sh_1, sh_2, sh_3 and a mix of the three shRNA-Runx2 plasmids (mix sh) delivered by C60-PEI nanoconjugates. The exposure of VIC for 14 days to HGOM induced an increase by 5.7-fold in ALP activity. After the second transfection of VIC with polyplexes in day 12, the ALP activity induced by HGOM, decreased by 35%, 26%, 40% and 50% following cell transfection with sh_1, sh_2, sh_3 and mix sh. Interestingly, the decreased ALP activity induced by VIC transfection with shRNA specific for Runx2 was maintained for 21 days, when a reduction of ~60% was obtained in VIC transfected with C60-PEI complexed with sh_1, sh_2, sh_3 and mix sh as compared to non-transfected VIC exposed to HGOM. The data correlate well with histochemical staining for the presence of ALP performed in the 14th day after two transfections with shRNA-Runx2 plasmids (Figure 5B). Together these results show that the downregulation of Runx2 determines a reduction in the ALP activity. 

## 4. Discussion

An important role in aortic valve calcification was shown to be played by VIC, that in pathological conditions acquire an osteoblast-like phenotype [32]. RNA interference (RNAi) might be a suitable technique for intervention in the post-transcriptional gene silencing of major players involved in the osteodifferentiation process of VIC. The advancement in clinical translation of RNAi therapeutics depends on the progress in the development of suitable siRNA/shRNA/miRNA carriers [33,34]. There were numerous efforts to develop proper nanocarriers for efficient delivery of siRNA/shRNA to different cell types [35,36]. Reports are showing the use of commercially available non-viral transfection reagents to deliver siRNA sequences targeting genes involved in VIC differentiation towards osteoblasts [37,38,39,40,41]. 

Due to the role played by the transcription factor Runx2 in the orchestration of the osteodifferentiation process of human VIC exposed to pro-osteogenic stimuli [42,43], we investigated whether nanoparticles able to deliver intracellularly the shRNA sequences targeting Runx2 (shRNA-Runx2) into VIC, in which the osteogenic differentiation has been activated, will halt or reverse the osteodifferentiation process. To efficiently deliver shRNA-Runx2 plasmid into VIC, we used the previously characterized C60-PEI nanoconjugates, able to wrap and protect plasmid DNA at N/P ratio higher than 5 [28]. The polyplexes C60-PEI/shRNA were physico-chemical characterized and the results indicated effective packing and protection of shRNA plasmid by C60-PEI nanoconjugates and, just as important, that they were not cytotoxic for cells. The viability of VIC is not significantly decreased by incubation with C60-PEI/shRNA plasmid nano-polyplexes at N/P ratio = 25, a fact that proposes the polyplexes at this N/P ratio as biocompatible, according to International Organization for Standardization, ISO 10993-5:2009 “Biological Evaluation of Medical Devices Part 5: Tests for in Vitro Cytotoxicity, 2009”. Moreover, we determined an efficient intracellular delivery of polyplexes C60-PEI/Cy3-labeled plasmid into VIC, as demonstrated by the intracellular presence of red fluorescent dots by fluorescence microscopy, and by flow cytometry measurements that revealed 80% of positive cells in FL3 channel. In general, as compared to the commercial transfection reagent, the use of C60-PEI as vectors for plasmid transfection in VIC has the advantage of a better transfection efficiency combined with low cytotoxicity. Together, our results showing that the highest transfection efficiency of VIC was obtained for C60-PEI/pEYFP plasmid polyplexes at N/P ratio of 25, corroborated with the viability assay data determined the choose of N/P ratio = 25 for further experiments of Runx2 silencing in VIC-committed to osteoblast-like cells. 

In this study, to induce osteoblast differentiation of human VIC, characteristic for diabetes, we exposed the cells to medium containing high glucose concentration (HG), osteogenic medium (OM) or a combination of the two (HGOM). There is evidence that diabetes can enhance the inflammatory response [44] and lipid accumulation in the valve, contributing to oxidative stress, the formation of advanced glycation end products and modification in calcium metabolism, accelerating thus the progression of valve calcification [45,46]. Hence, we investigated whether the high glucose concentration adds a synergistic effect to osteogenic factors and determines the increased expression of osteogenic proteins.

In our experiments, human VIC, isolated from noncalcified aortic valves or portions of the cusp of aortic valves, have been used. We investigated the phenotypic transition of human VIC to osteoblast-like cells induced by their exposure to HG, OM or HGOM for different periods from 2 to 21 days. A time-dependent increase in α-SMA protein expression was determined in VIC cultured in OM and HGOM, pointing to the VIC activation to a myofibroblast phenotype. At 21 days, a slight decrease in α-SMA expression was obtained, a result in line with previous reports that showed a decrease in α-SMA protein expression with the phenotypic transition from VIC-myofibroblast to VIC-osteoblast [47]. The differentiation of VIC into osteoblasts resembles physiological osteogenesis and is mainly controlled by Runx2, a key osteoblast-specific transcription factor. The level of Runx2 protein expression is significantly higher in the case of VIC’s exposure, for a period up to 14 days to HGOM, as compared to HG or OM. These results suggest that the combination of high glucose concentrations with osteogenic factors has a synergistic effect on Runx2 levels, determining a significant increase in the Runx2 expression (by 3.5-fold) as compared with OM (by 2.5-fold). A possible explanation could be the multiple signaling pathways that converge to the Runx2 transcription factor expression [48] that can be distinctly activated by HG, over that stimulated by osteogenic factors. The time-course experiments revealed that the Runx2 level peaked at 14 days of activation with either HGOM or OM. These data confirm the studies showing the highest expression of Runx2 at 14 days of VIC incubation in medium containing osteogenic factors [49,50]. 

The protein expression of other osteogenesis markers, namely ALP and BSP showed a significant increased value in VIC exposed to OM and HGOM. The expression of these osteogenic proteins was higher in OM or HGOM-treated VIC when compared to HG, clearly showing that alone, high glucose concentration was not sufficient to increase their level in VIC, which was significantly increased by the exposure of cells to OM or a combination of high glucose concentration with osteogenic factors. Altogether, the VIC’ activation experiments show that HGOM induces the transition of VIC to myofibroblast, and, at longer incubation time, the osteodifferentiation of VIC [47].

To investigate whether the pathological process of osteodifferentiation in human VIC can be stopped by Runx2 silencing, we used, for VIC transfection, C60-PEI/shRNA-Runx2 plasmid nano-polyplexes formed with three sequences of shRNA targeting Runx2. We report here that the mRNA and protein expression of Runx2 is significantly decreased at 48 h after the transfection of HGOM-treated VIC with each of the three shRNA-Runx2 sequences used to form C60-PEI/shRNA-Runx2 nano-polyplexes. Furthermore, the C60-PEI/shRNA-Runx2 nano-polyplexes-mediated knock-down of Runx2 in osteoblast-committed VIC reduced the expression of osteogenic proteins ALP, OSP, BSP, and BMP-4, confirming the previous studies showing the major role played by Runx2 in controlling the expression of osteoblast marker genes [14]. Remarkably, the biological effect of Runx-2 reduction was sustained for more than one week. Thus, at 21 days, in VIC exposed to HGOM and receiving two treatments of shRNA-Runx2 carrying nanoparticles (in the 5th and 12th days), the ALP activity was significantly reduced by 60% as compared with that determined in non-transfected VIC exposed to HGOM. This fact suggests that the osteogenic shift of VIC may be inhibited by nanoparticles able to efficiently deliver intracellularly shRNA sequences specific for Runx2. So, the impeding of VIC’ activation and osteodifferentiation using nanocarriers of shRNA-Runx2 may provide a new therapeutic strategy to modulate CAVD progression. To the best of our knowledge, this is the first report on the use of nanocarriers to deliver shRNA targeting Runx2 to suppress the osteogenic response of human VIC to high glucose concentration and osteogenic factors. The new data may provide a strategy to control, by a non-surgical therapy, CAVD progression in diabetes.

The results motivate further in vivo testing of this proof of concept using C60-PEI/shRNA-Runx2 polyplexes encapsulated into targeted nanoparticles, designed to recognize molecular targets expressed by diseased aortic valve, by coupling specific ligands to their surface.

## 5. Conclusions

In this study we showed that the delivery of Runx2-shRNA plasmids by nano-polyplexes down-regulates the expression of proteins involved in osteodifferentiation of human valvular interstitial cells in diabetic and pro-osteogenic conditions. The data suggest that the silencing of Runx2 could represent a novel strategy to impede the osteoblastic phenotypic shift of VIC and the ensuing progress of CAVD.
